# Reply to Kovacs, F.M.; Seco-Calvo, J. Limitations of a Cross-Sectional Correlation Study. Comment on “Elabd et al. Prediction of Back Disability Using Clinical, Functional, and Biomechanical Variables in Adults with Chronic Nonspecific Low Back Pain. *J. Clin. Med.* 2024, *13*, 3980”

**DOI:** 10.3390/jcm13195774

**Published:** 2024-09-27

**Authors:** Omar M. Elabd, Paul A. Oakley, Aliaa M. Elabd

**Affiliations:** 1 Department of Orthopedics and Its Surgeries, Faculty of Physical Therapy, Delta University for Science and Technology, Gamasa 35712, Egypt; 3omarel3abd@gmail.com; 2 Department of Physical Therapy, Aqaba University of Technology, Aqaba 771111, Jordan; 3 Private Practice, Newmarket, ON L3Y 8Y8, Canada; docoakley.icc@gmail.com; 4 Kinesiology and Health Science, York University, Toronto, ON M3J 1P3, Canada; 5 Basic Science Department, Faculty of Physical Therapy, Benha University, Benha 13511, Egypt

Herein, we reply to comments made by Kovacs and Seco-Calvo [1] regarding our recent cross-sectional correlational study which aimed to predict lower back disability using clinical, functional, and biomechanical variables in adults with chronic nonspecific lower back pain [2]. We appreciate the comments made and address each.

Regrading point 1 [1], the criticism of not including psychosocial variables, we do acknowledge the potential limitations of not including psychosocial and work-related factors. We do argue, however, that since recent systematic reviews and meta-analyses have shown psychosocial variables to have inconsistent and weak influences on pain and disability in chronic musculoskeletal pain subjects [3,4,5], our results would have remained consistent if we included such variables. Kovacs et al. [1] also critiqued the sample size as being small; however, we believe our sample size of 100 was sufficient, particularly since we determined a required size of *n* = 91 using a moderate effect size (Cohen’s *f*^2^ = 0.15) [6] with an 80% power and 95% significance level (*p* < 0.05) for the multiple regression analysis [7].

Criticism point 2 was the lack of an emphasis on the multivariate regression prediction model that showed only pain intensity and back muscle endurance as being statistically significant as opposed to all of the clinical, functional, and biomechanical variables showing statistical significance in the univariate regression prediction models [1]. We believe these results were emphasized in our paper both in the Results and Discussion sections. Moreover, as highlighted in the Discussion section, it is not surprising, as most items on the Oswestry disability index focus on clinical suffering during functional activities, particularly those requiring the constant contraction of the trunk extensor muscles.

The third criticism was that since our study was a correlational cross-sectional design, only correlations and not causations could be claimed [1]. We do not see any contradiction regarding our work, as our results relied on multiple regression models and not on correlation analysis alone. Importantly, both are different [8]. Correlation is a statistical measure that determines the association or co-relationship between two or more variables. Regression determines whether an independent variable really affects the dependent variable and estimates the magnitude of that effect. Thus, in general, prediction and causal effects can be estimated using regression. Multiple regression is a step beyond simple regression. Multiple regression includes two or more independent variables (sometimes called predictor variables) in the model rather than just the one that is included in the simple regression.

In the last criticism, building from the last, Kovacs et al. [1] argue that the results from our study “are not appropriate to support, challenge or modify current usual clinical practice”, and that our results fail to emphasize that a multidisciplinary approach needs to be included in the patient’s clinical assessment. We believe our results provide important contributions to current clinical practice, as our results reveal that each of the measured independent variables (clinical, functional, and biomechanical) were significant predictors of back disability; moreover, among all of the measured variables, the results highlight the significant contribution of the clinical variables (pain intensity and back extensor endurance) in the multi-regression model for the prediction of disability. The basis of the criticism highlights the difference between correlation and regression analysis; the latter enables one to predict, and, therefore, we argue does point to the fact that not only a multimodal treatment approach, but a multi-modal assessment approach should be taken to better document the all-encompassing aspects within the clinical, functional, and biomechanical domains that intertwine and contribute to non-specific lower back pain. Of course, we look forward to future research corroborating our findings.
